# A121 CROSS-SECTIONAL STUDY OF RESIDENT PHYSICIAN KNOWLEDGE AND PERCEPTIONS REGARDING NON-ALCOHOLIC FATTY LIVER DISEASE IN CANADA

**DOI:** 10.1093/jcag/gwad061.121

**Published:** 2024-02-14

**Authors:** K Zhu, M Jogendran, Y Zhang, T Hussaini, D Chahal, E Yoshida

**Affiliations:** The University of British Columbia Faculty of Medicine, Vancouver, BC, Canada; Queen's University Department of Medicine, Kingston, ON, Canada; The University of British Columbia Faculty of Medicine, Vancouver, BC, Canada; The University of British Columbia Faculty of Medicine, Vancouver, BC, Canada; The University of British Columbia Faculty of Medicine, Vancouver, BC, Canada; The University of British Columbia Faculty of Medicine, Vancouver, BC, Canada

## Abstract

**Background:**

Non-alcoholic Fatty Liver Disease (NAFLD) is a prevalent condition affecting approximately 25% of the Canadian population and is projected to continue increasing. Given the increasing prevalence and complications of NAFLD, it is imperative for future primary care physicians to possess a thorough understanding of NAFLD to enhance patient care.

**Aims:**

To assess the knowledge and perceptions of primary care resident physicians in Canada concerning NAFLD.

**Methods:**

We conducted a nationwide cross-sectional survey of resident physicians in primary care specialties to assess their knowledge of NAFLD. Additionally, we evaluated resident perceptions regarding the importance of NAFLD and their exposure and familiarity with the condition. "Reasonable knowledge" was defined as correctly answering more than 50% of the questions. We assessed associations using χ^2^ testing and multiple logistic regression analysis.

**Results:**

We received 413 responses, with 252 (61%) from family medicine residents and 161 (39%) from internal medicine residents. Among the respondents, 90% considered NAFLD an important public health issue, but only 7% felt they had adequate exposure to the condition, and 94% expressed a need for more teaching. 59% indicated that they were only slightly familiar or unfamiliar with NAFLD. The majority of respondents correctly identified diabetes (90%), dyslipidemia (96%), and obesity (97%) as risk factors for NAFLD, while fewer recognized obstructive sleep apnea (53%), hypothyroidism (27%) and hypopituitarism (14%) as risk factors. Cardiovascular disease and cirrhosis were reported as the most common causes of mortality from NAFLD by 37% and 35% of respondents, respectively. Up to 59% of respondents reported that non-alcoholic steatohepatitis can be diagnosed through imaging or a blood test. Respondents demonstrated a strong understanding of nonpharmacologic therapies, but only 11% recognized that there are no approved medications for NAFLD.

Overall, 35% of the respondents displayed a reasonable knowledge of NAFLD. In univariate analysis, factors associated with greater NAFLD knowledge included internal medicine residency (P=0.001), higher post-graduate year (P=0.003), prior GI or hepatology rotations (P=0.003), and subjective familiarity with NAFLD (Pampersand:003C0.001). However, only higher post-graduate year (P=0.048) and subjective familiarity (P=0.003) remained statistically significant in multivariate analysis.

**Conclusions:**

Resident physicians perceive NAFLD as an important health issue but significantly lack exposure and knowledge about the condition. Further emphasis and education are needed to bridge these knowledge gaps and improve patient outcomes.

**Funding Agencies:**

None

Endoscopy, Technology & Imaging

